# PCA-based detection of phosphorous deficiency in wheat plants using prompt fluorescence and 820 nm modulated reflection signals

**DOI:** 10.1371/journal.pone.0286046

**Published:** 2023-05-24

**Authors:** Yousra El-Mejjaouy, Laila Belmrhar, Youssef Zeroual, Benjamin Dumont, Benoît Mercatoris, Abdallah Oukarroum

**Affiliations:** 1 AgoBioSciences, Plant Stress Physiology Laboratory, University Mohammed VI Polytechnic (UM6P), Benguerir, Morocco; 2 Biosystems Dynamics and Exchanges, TERRA Teaching and Research Centre, Gembloux Agro-Bio Tech, University of Liege, Gembloux, Belgium; 3 Pant Sciences / Crop Science, TERRA Teaching and Research Centre, Gembloux Agro-Bio Tech, University of Liege, Gembloux, Belgium; 4 High Throughput Multidisciplinary Research Laboratory, University Mohammed VI Polytechnic (UM6P), Benguerir, Morocco; United Arab Emirates University, UNITED ARAB EMIRATES

## Abstract

Phosphorus deficiency induces biochemical and morphological changes which affect crop yield and production. Prompt fluorescence signal characterizes the PSII activity and electron transport from PSII to PSI, while the modulated light reflection at 820 (MR 820) nm investigates the redox state of photosystem I (PSI) and plastocyanin (PC). Therefore, combining information from modulated reflection at 820 nm with chlorophyll a fluorescence can potentially provide a more complete understanding of the photosynthetic process and integrating other plant physiological measurements may help to increase the accuracy of detecting the phosphorus deficiency in wheat leaves. In our study, we combined the chlorophyll *a* fluorescence and MR 820 signals to study the response of wheat plants to phosphorus deficiency as indirect tools for phosphorus plant status characterization. In addition, we studied the changes in chlorophyll content index, stomatal conductance (g_s_), root morphology, and biomass of wheat plants. The results showed an alteration in the electron transport chain as a specific response to P deficiency in the I-P phase during the reduction of the acceptor side of PSI. Furthermore, P deficiency increased parameters related to the energy fluxes per reaction centers, namely ETo/RC, REo/RC, ABS/RC, and DIo/RC. P deficiency increased the values of MR_min_ and MR_max_ and decreased ν_red_, which implies that the reduction of PSI and PC became slower as the phosphorus decreased. The principal component analysis of the modulated reflection and chlorophyll a fluorescence parameters, with the integration of the growth parameters as supplementary variables, accounted for over 71% of the total variance in our phosphorus data using two components and provided a reliable information on PSII and PSI photochemistry under P deficiency.

## Introduction

Phosphorus is a vital element in photosynthesis, which affects in e.g, ATP and NADPH formations, as well as in sugar phosphates and phospholipids, which are strongly involved in photosynthesis [1]. Consequently, phosphorus deficiency affects crop yield and production. Phosphorus (P) is a relatively immobile nutrient in soil because of its great adsorption to the soil matrix, and more than 80% amount is present in an unavailable form for plant uptake [2]. Thus, the application of P as fertilizer might be necessary to ensure plant growth and development. Plants absorb phosphorus in the form of orthophosphate H_2_PO_4_^−^, HPO_4_^2-^ [3]. However, in recent decades, water-soluble phosphorus fertilizers, called polyphosphates (PP), have been used in different agricultural systems [4]. Polyphosphates are anionic linear polymers of orthophosphate linked by hydrogen phosphate bonds. Supplied PP in the nutrient solution has been reported to enhance plant growth and its development, such as earlier flowering [5]. In sweet pepper fruits, PP increased the photosynthetic capacity and the total yield [6].

Phosphorus deficiency has been reported to have a direct effect on plant growth and productivity, impacting both shoot and root compartment [7]. Under limited P supply, plants develop multiple responses. They can proceed by decreasing the growth rate of aboveground biomass—therefore increasing the growth per unit of P uptake—or by inducing changes in their root architecture to increase mobilization of soil P reserves [8]. Sitko et al. [9] observed low photosynthetic and transpiration rates, and a maximum inhibition of stomatal conductance in P-deprived plants compared to Ca, K, Mg deficiencies. Decrease in stomatal conductance and photosynthetic and transpiration rates was also reported by Veronica et al. [10] for all rice genotypes grown at low P concentrations. Phosphorus stress disturbs the photosynthetic pigment production such as chlorophyll and anthocyanin. P deficient plants have higher anthocyanin content that causes generally an enhancement of red colouration [11]. Phosphorus stress in plants also causes a change in their optical and spectroscopic properties such as fluorescence [12]. Under solar radiation, chlorophylls in the chloroplasts absorb a significant fraction of the light, transmit another fraction, and reflect the third. The absorbed part may be involved in photochemical reactions related to photosynthesis, or it may be dissipated in the form of heat or chlorophyll fluorescence emission [13]. Depending on the wavelengths, these proportions vary depending on the regulation dynamics and the chemical and physical composition of the leaves. Therefore, understanding the fluorescence emission of leaves in response to phosphorus stress can provide an accurate assessment of physiological state of the plant and its phosphorus status.

It was suggested that nutrient deficiency induces a specific response in plants, as it affects the electron transport chain compounds associated with the photosystem II (PSII) donor or acceptor sides, or with the photosystem I (PSI) acceptor side [14]. Additionally, they have demonstrated that nutrient deficiency reduces the PSII photochemical efficiency and the fraction of active reaction centers that participate in non-photochemical dissipation of the absorbed energy in the PSII antenna. Phosphorus deficiency stress was found to affect the electron transport at photosystem I (PSI), which had led to the alteration of the I-step of the fluorescence transient, called also the OJIP transient [15]. In addition to the analysis of the OJIP curve shape, chlorophyll *a* fluorescence parameters were also used to study P deficiency effect on photosynthetic activity. According to Veronica et al. [10], low phosphorus increased the nonphotochemical quenching while decreasing the effective PSII quantum yield, electron transport rate, and the coefficient of photochemical quenching. Kalaji et al. [14] investigated the effect of P deficiency on photosynthetic parameters in tomato plants. P deficiency has affected photosynthetic parameters related to energy dissipation such as φ_Do_ and DIo/RC to protect nutrient-deficient leaves from excessive absorbed light energy and photo-oxidative damage. Simultaneous chlorophyll fluorescence OJIP transients and 820 nm-modulated reflection measurements allow to explore the photosynthetic electron transport chain reduction and the interaction between PSII and PSI [16], which provides useful information about the photosynthetic activity. The prompt chlorophyll fluorescence induction provides information regarding the PSII reactions, while the electron flow from the plastoquinone to the PSI is studied by measuring the modulated reflection at 820 nm [17, 18]. Chlorophyll *a* fluorescence transient (OJIP transient) has been widely used to study the photosynthetic electron transport performance and related photosynthetic processes in stressed plants [19–23].

The simultaneously recorded signals of photoinduced prompt chlorophyll fluorescence (PF) and modulated reflection (MR) at 820 nm were used to assess the effect of different stressors on the plant physiological state, namely drought stress, nitrogen deficiency, soil salinity, temperature, and zinc application [16, 17, 24, 25]. However, few recent research was conducted to simultaneously monitor PSI and PSII performances for phosphorus deficiency. P deficiency affects electron flow from the plastoquinone pool to PSI probably due to a fast and strong generation of Photosynthetic Control [26]. The chlorophyll fluorescence measurements have been proved to characterize the PSII activity and electron transport from PSII to PSI and to present great sensitivity. However, it was assumed that complementary information about PSI given by MR signal and its parameters can offer a clear and complete understanding of the effects of phosphorus on photosynthetic apparatus, and enhance the accuracy of phosphorus deficiency detection, in addition to the information provided by chlorophyll fluorescence measurements.

In this study, the impact of phosphorus deficiency on the growth of wheat plant (*Triticum durum L*.*)* was investigated. The way in which P deficiency affects PSII and PSI photochemistry was analyzed in vivo, using two signals measured simultaneously, namely chlorophyll fluorescence transient and modulated reflection at 820 nm (MR). The parameters related to the chlorophyll *a* fluorescence and MR signals, along with other physiological and morphological measurements, were utilized to investigate their potential in detecting phosphorus deficiency in wheat leaves.

## Materials and methods

### Plant material, fertilisation, and experimental design

Wheat seeds (Karim variety—*Triticum durum L*.) were sown in peat and irrigated every day for six days with distilled water, under controlled conditions with a temperature of 25°C, light intensity of 104 μmol m^-2^ s^-1^, photoperiod of 12-hour light/12-hour dark, and humidity maintained at 60%. We transplanted the seedlings into Hoagland solution specifically formulated for wheat seedlings, which was changed each 3 days. The macronutrients in the solution were as follows: (in mM): 0.5 Cl, 2.5 Ca, 7.5 N, 3.0 K, 1.0 S, 1.0 Mg, and P (see here after)—as well as the following micronutrients: (in μM) 60 Fe, 19 B, 0.92 Zn, 3.64 Mn, 0.47 Cu, and 0.01 Mo.

Wheat plants were hydroponically cultivated using two different doses of P, to create a well-nourished reference treatment and a deficient treatment. Under the full P dose, the plants were treated with 0.5 mM, and under the P1/2 dose, plants were treated with 50% of their phosphorus’ need (i.e. 0.25mM). Three sources of P, provided from three water-soluble fertilizers forms, were tested: two orthophosphates (Ortho-A and Ortho-B), and a polyphosphate (Poly-B). In addition to the two phosphorus concentrations, a control treatment (C) was added, in which no phosphorus was added to the nutrient solution. The experiment was conducted in a completely randomized block design, all the treated plants were grown simultaneously in identical conditions and each treatment was replicated three times. For each replicate, 12 seedlings were transplanted, for a total of 252 plants ((3 P form x 2 P doses + Control) x 3 replicates). Measurements were taken after one and six weeks of growth in the nutritive solutions.

### Chlorophyll content index

Chlorophyll content index (CCI) values were measured using a non-destructive portable chlorophyll meter (CL-O1, Hansatech instruments). CCI data were acquired from the middle part of the mature wheat leaves of all plants, after having been kept 1 min in the dark. For each treatment, the CCI measurements were taken on at least 6 independent leaves (i.e., from six plants) per replicate.

### Stomatal conductance

The stomatal conductance (g_s_) of all treatments was determined using a leaf porometer (SC-1 Leaf porometer Decagon Devices, Inc.). Measurements were made in the morning. At least 6 independent measurements (six plants) were taken, and the average was used for analysis.

### Root morphology

After six weeks of growth, 10 roots were collected and spread with water in a plastic box. The Epson Perfection LA2400 scanner was used to image the roots. The obtained images were then analyzed using WinRHIZO software (Regent Instructions, Quebec, Canada) to measure root length, root surface area, root volume and tips.

### Biomass

After six weeks of plant growth in nutritive solution, 6 roots and shoots were chosen among the plants used for roots scanning in order to determine the biomass. The samples were kept in an oven for at 70°C for 2 days to measure the dry mass.

### Phosphorus content analysis

Elemental concentration of P was analyzed on a dry-mass basis using Inductively Coupled Plasma Optical Emission Spectrometry (Agilent 5110 ICP-OES, USA). The dried samples were divided into 3 subsamples to get the minimal dry mass required for the chemical analysis in order to quantify P content.

### Prompt fluorescence and modulated reflection at 820 nm

M-PEA-1 instrument (Hansatech Instruments Ltd, King’s Lynn, UK) allows to determine the prompt chlorophyll fluorescence and modulated reflection at 820 nm measurements simultaneously. Before taking the measurements, the plants were kept in darkness for 10 min. The M-PEA instrument has two types of LEDs emitting at 627±10 nm and 820±25 nm, respectively. During the fluorescence measurements we used the red actinic illumination of 5000 μmol photon m^−2^ s^−1^ for 10s. The modulated reflection at 820 nm is represented by the MR/ MR_o_ ratio, where MR_o_ is the value at the onset of the actinic illumination; this ratio is complementary to the ratio (I_abs_/I_inc_)820 nm, where I_inc_ is the incident light and I_abs_ is the light absorbed by the sample at 820 nm [27]. The MR_t_/MR_o_ ratio, where MR_t_ is the modulated 820-nm reflection signal recorded at each time step during the 1s illumination, and MR_o_ is the value at the beginning of actinic illumination, measured at 0.3 ms. The MR_min_ and MR_max_ represents the minimal and maximal MR_t_/MR_o_ values, which were obtained at 20 ms and 300 ms, respectively.

Additionally, the ν_ox_ and ν_red_ values, which were used in the PCA analysis, were calculated using the following formulas:

$$\nu_{\text{ox}}=\left(MRmin-MRo\right)/\left(20-0.3\right)$$ (1)

$$\nu_{\text{red}}=\left(MRmax-MRmin\right)/\left(300-20\right)$$ (2)

They represent the rates of P700+PC oxidation and the subsequent re-reduction, respectively (see e.g., [28]). When the oxidation and the re-reduction rates are equal, a transitory steady state occurs as the min of MR_t_/MR_o_ (MR_min_).

The O-J phase is referred to as the photochemical phase of the prompt chlorophyll fluorescence curve, as the J amplitude increases with the light intensity [29–31]. This photochemical phase provides information on antenna size and PSII reaction centres connectivity [32]. The J-P phase is named the thermal phase [29]. The J-I phase was proposed to be linked with the reduction of the PQ-pool, while the I-P phase was associated with electron transport through PSI [33, 34].

The effect of P deficiency on OJIP characteristics was investigated by normalizing the fluorescence curves obtained during the IP phase to the fluorescence reported during the I stage (F_I_) using the formula F_t_/F_I_. The curves were also double normalised between I and P phase using the fluorescence at I and P steps (F_I_ and F_M_, respectively) [14], following V_IP_ = (F_t_ − F_I_)/ (F_M_ − F_I_), which allow to evaluate changes in PSI as changes in the V_IP_ follow changes in PSI content. In addition, the relative variable fluorescence at J-step (V_J_) and I-step (V_I_) were calculated using, respectively, the following equations:

$${\text{V}}_{\text{J}}=\left({\text{F}}_{\text{J}}-{\text{F}}_{\text{O}}\right)/\left({\text{F}}_{\text{M}}-{\text{F}}_{\text{O}}\right)$$ (3)

and

$${\text{V}}_{\text{I}}=\left({\text{F}}_{\text{I}}-{\text{F}}_{\text{O}}\right)/\left({\text{F}}_{\text{M}}-{\text{F}}_{\text{O}}\right).$$ (4)

To better investigate the effect of P supply on chlorophyll fluorescence transients, the differential curves (ΔV_t_) were calculated by subtracting the double normalized fluorescence values between F_O_ and F_M_ (V_t_), measured in plants growing in sufficient P concentration(P) from those recorded in plants growing in low P concentrations (Control and P1/2) [35]:

$${\text{V}}_{\text{t}}=\left({\text{F}}_{\text{t}}-{\text{F}}_{\text{O}}\right)/\left({\text{F}}_{\text{M}}-{\text{F}}_{\text{O}}\right).$$ (5)

$$\Delta {\text{V}}_{\text{t}}={\text{V}}_{\text{t}\;(\text{Control}\;\text{and}\;\text{P}1/2)}-{\text{V}}_{\text{t}(\text{P})}.$$ (6)

The OJIP parameters were averaged for each P concentration, normalized to the values from the control treatment, and presented as radar plots. Due to the small number of observations (n = 20), the OJIP parameters were reduced to 12 parameters based on the radar charts result and our previous study [21]. Then, ones of the highly correlated fluorescence parameters were chosen for the analysis. MR changes were represented only by ν_ox_ and ν_red_ since they are highly correlated to MR_min_ and MR_max_. The selected variables were used to conduct a principal component analysis (PCA), in addition to plant growth parameters that were added as supplementary variables. The description of JIP-test and modulated reflection parameters is given in S1 Table.

### Statistical analysis

Statistical analysis ANOVA (for P<0.05) was performed using SPSS data processing software (SPSS 20.0) considering three independent replicates per treatment. Normal distribution of the data was checked, and the homogeneity of variances was tested operating the Levene’s test with p-value equal to 0.05. The two-way analysis of variance was performed to investigate the interaction of fertilizer form vs phosphorus level (p < 0.05). If significant interactions were found, the treatments were intercompared and ranked using Tukey post-hoc test at a 0.05 confidence level. The principal component analysis was conducted in RStudio using the measurements of the sixth after transplantation and all the variables were centered and scaled before the analysis.

## Results

### Chlorophyll content index, stomatal conductance, and biomass

**Fig 1:**
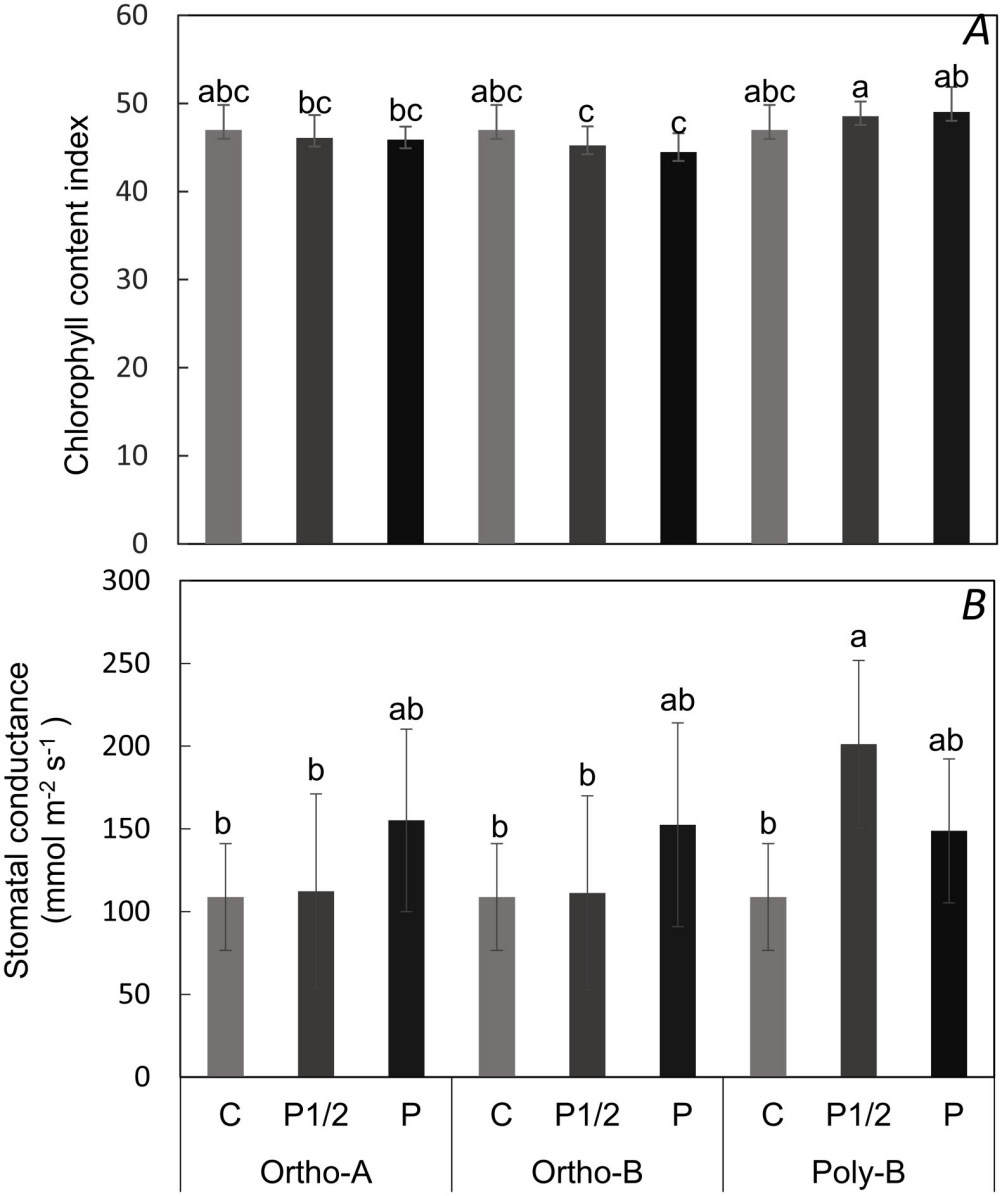
Effect of P fertilizer form combined with P level on the two parameters. (A) chlorophyll content index (B) and stomatal conductance (B). Comparison of results using the Tukey test are reported on the graph using letters; identical letters indicate that results belong to the same statistical group.

Fertilizer form and phosphorus level was found to have a significant impact on the chlorophyll content index (CCI) after six weeks following transplantation. The polyphosphate fertilizer form at the concentrations P1/2 and P had the highest values of CCI followed by the control treatment. Regardless of the P level used, the ortho-A and the ortho-B fertilizer forms had the lowest values values of CCI (Fig 1A).

Six weeks after transplantation, the interactive effect of fertilizer form and applied phosphorus amount had a significant effect on the stomatal conductance (g_s_). In Fig 1B, the stomatal conductance showed a slight decrease to 120 mmol m^-2^ s^-1^ in deficient leaves grown in the control and P1/2 treatments control, provided by the ortho-A and ortho-B fertilizer forms. Poly-B fertilizer form seems to enhance stomatal conductance in plants growing at low P (P1/2) and it was 35% higher compared to plants growing in sufficient P (P).

**Fig 2:**
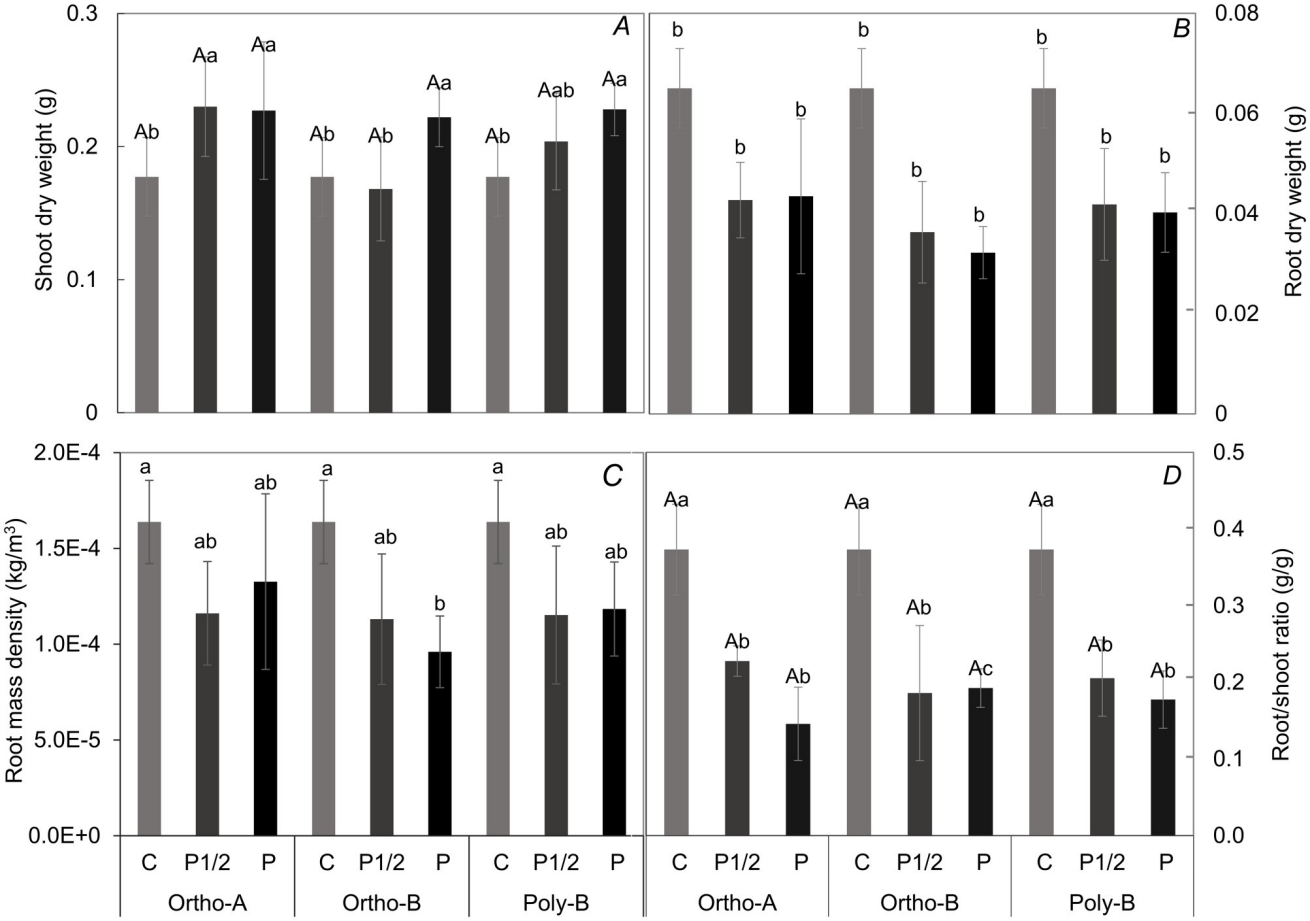
Growth parameters of wheat seedlings after 6 weeks of growth. (A) Shoot dry mass, (B) root dry mass, (C) root mass density, and (D)root/shoot ratio. (A; effect of fertilizer form) and (a and b; effect of P level).

The fertilizer form had no effect on shoot dry mass and shoot/root ratio (Fig 2A and 2D). Excluding the Ortho-A fertilizer form, the control plants had the lightest shoots of 0.18 g and the sufficient P treatment enhanced the shoot dry mass. On the other hand, an interactive effect of fertilizer form and P level was observed on root dry mass and mass density (Fig 2B and 2C). The control plants showed the highest ratio value of 0.39 and the highest value of root mass density (0.16 g.cm^-3^), whereas there was no significant difference between the P1/2 and P treatments.

### Root morphology

**Fig 3:**
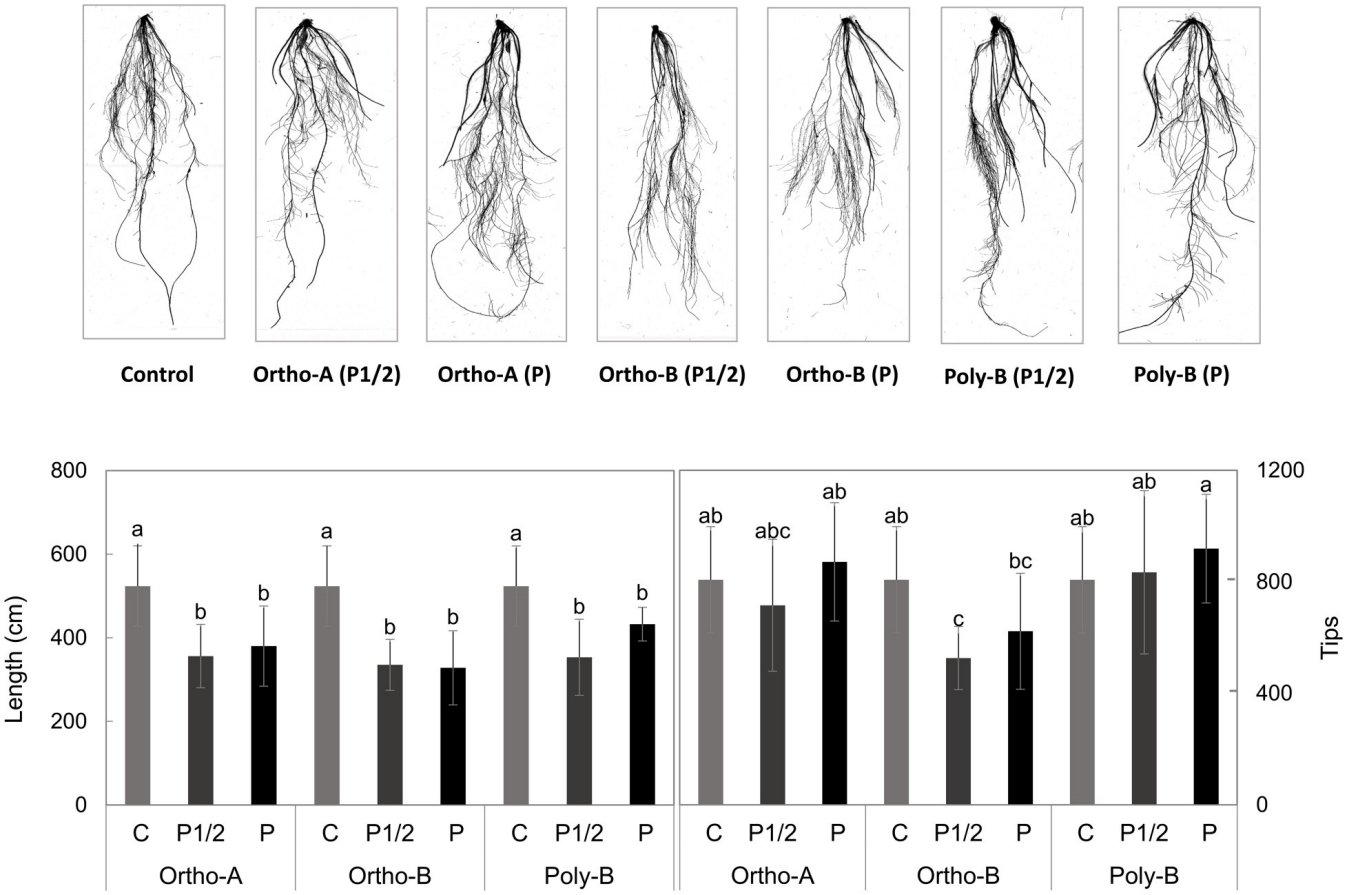
WinRhizo root images of the studied treatments and their effects on root parameters. The measurements of root morphology parameters, length and tips, were taken at 6 weeks after transplantation into Hoagland solution. The treatments are: fertilizer forms: Ortho-1, Ortho-B, and Poly-B, combined with two doses: 50% of P needs (P1/2), 100% covering plant P needs (P), and P deficient treatment as a control (C).

Length and tip parameters of the root morphology were analyzed and showed in Fig 3. Both parameters were affected by the interaction of fertilizer form and P level, this effect was more prominent on root tips than on length. The absence of phosphorus increased total root length compared to other treatments (Fig 3A). Four groups were distinguished for tips parameter, the highest value was observed in P treatment provided from poly-B fertilizer while the lowest was recorded by the ortho-B at P deficient treatment (P1/2) (Fig 3B).

#### Prompt fluorescence and modulated reflection signals

**Fig 4:**
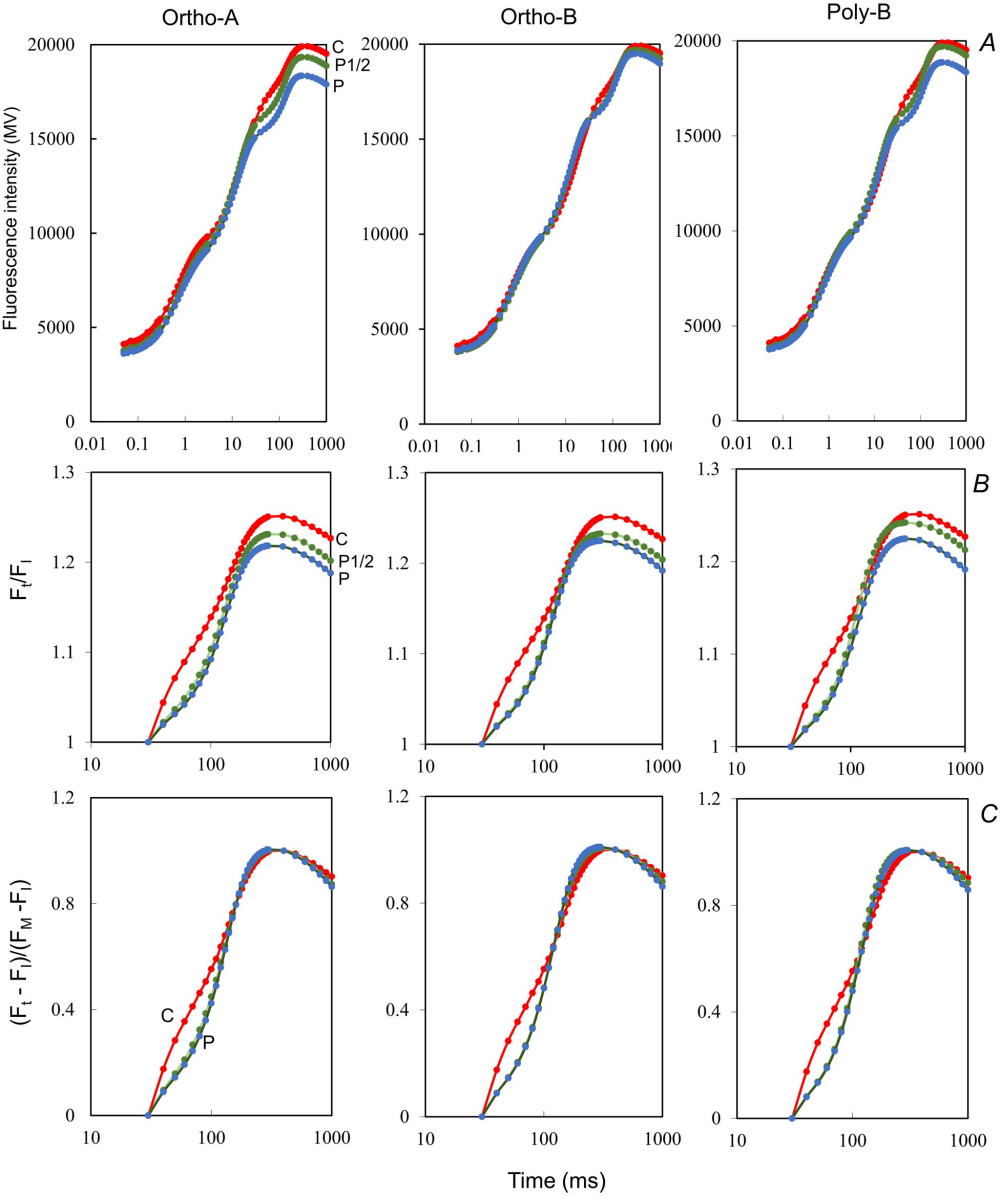
Chlorophyll a polyphasic fluorescence rise O-J-I-P of plants, at 6 weeks after transplantation. (A) The OJIP transients are plotted on logarithmic scale and (B) the relative (F_t_/F_I_) ratio are presented for a single turn-over phase (I-P). (C) The relative variable fluorescence of the different fluorescence transients, calculated as V_t_ = F_t_ − F_I_ / F_M_ − F_I_, are plotted for a single turn-over phase (I-P).

The prompt chlorophyll fluorescence measured after 6 weeks in all plants showed a typical transient OJIP form. Under low P, the O-J and J-I phases seemed to be unaffected by the P treatment. However, plants of the nutritive solution with low P concentration showed differences in the rise kinetics reported at I-level and during I-P phase (Fig 4A).

F_IP_ amplitude (Fig 4B) was greater in plants growing without P and its shape was distinguishable from plants growing at low or sufficient P concentration. The slope of the IP rise phase in plants growing in low or sufficient P was steeper than it was in plants growing without P (Fig 4C). For plants grown in sufficient or low P concentration, the IP phase of the OJIP curve displayed a sigmoidal curve which tended to disappear in plants growing in nutritive solution without P (Fig 4A–4C).

**Fig 5:**
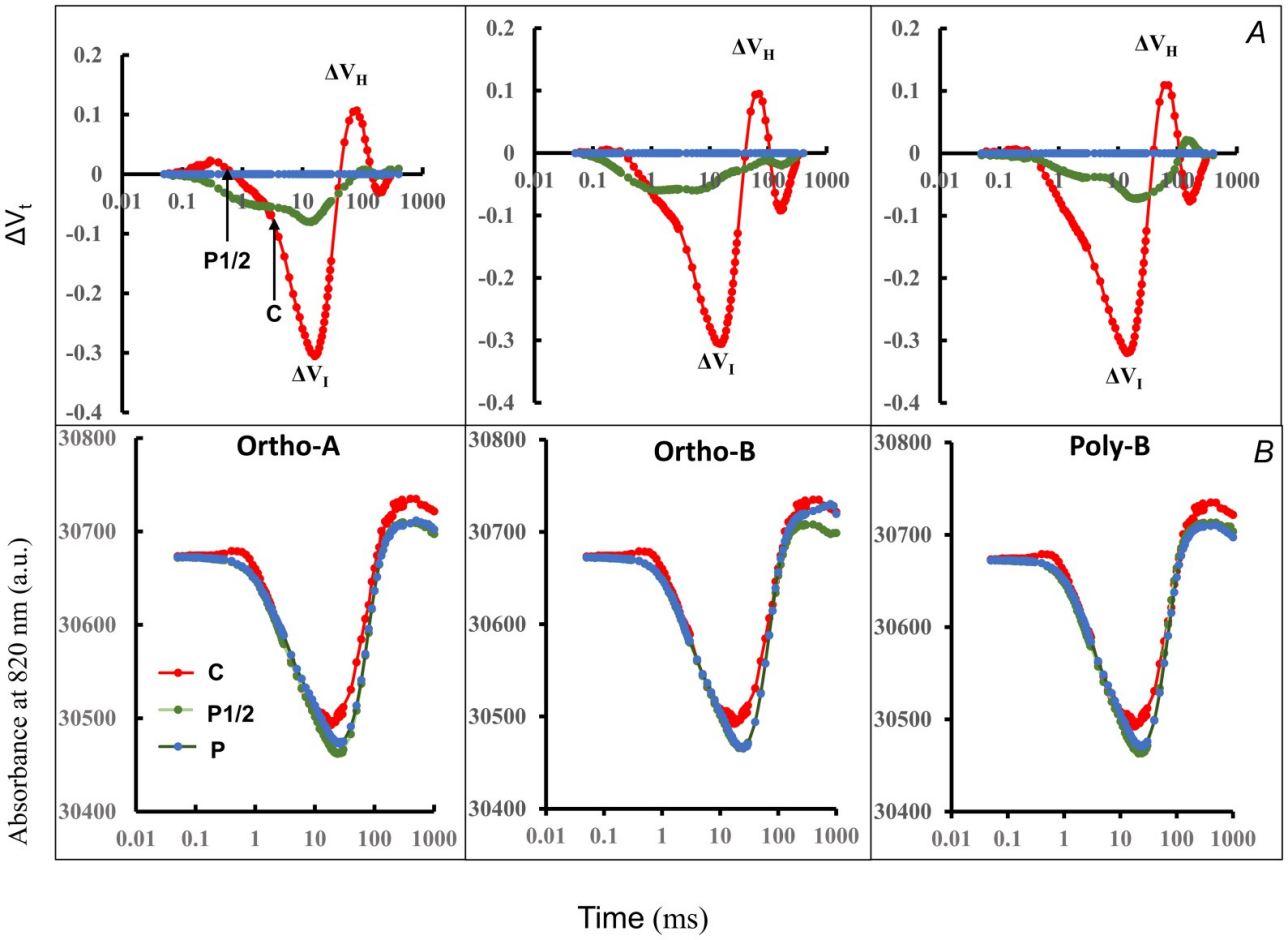
Changes in differential chlorophyll fluorescence curves modulated reflection at 820 nm. (A) Differential chlorophyll fluorescence normalized between O and P (ΔV_t_) and (B) changes in modulated reflection at 820 nm in leaves of seedlings grown for six weeks in different P concentrations and fertilizer forms. The MR820 curve is the average of 5 measurements (n = 5). ΔV_t_ were calculated by subtracting the double normalized fluorescence values between F_O_ and F_M_ (V_t_), measured in plants growing in sufficient P treatment from those recorded in plants growing in low P concentrations (C and P1/2) using the formula ΔV_t_ = V_t (Control and P1/2)_ -V_t(P)_.

Changes in OJIP transients were also defined by the difference in variable fluorescence curves ΔV_t_ (Fig 5). The ΔV_t_ curves were calculated at the beginning of treatment (1week) and after six weeks of treatments and results are reported at Fig 5. At the beginning of treatment (Fig 5A), analysis of the fluorescence transients revealed that the effects of P deficiency occurred over the whole OJIP phases. However, after 6 weeks of treatment, changes were evident in the J-P phase. Two bands with peaks appeared at 30 ms (I step) and at 100 ms (H band). The difference kinetics showed hidden bands between the steps O, J, I, and P without subtraction from control. We should note that plants growing in Poly-B with 50% P concentration (P1/2) seems to be tolerant to P deficiency at the beginning of treatment, with the exhibition of positive bands. To a lower extent, plant growing in Ortho-B à 50% P concentration seemed less impacted by P-deficiencies, as compared to plant growing in Ortho-A at similar concentration. However, after 6 weeks of treatment (Fig 5A), ΔV_t_ showed the same patterns for all forms, respectively under each dose. Changes in the 820nm modulated reflection were used in this study to probe electron flow through PSI. Fig 5B shows the kinetic changes of MR induced by red actinic light of 5000 mmol photons m^-2^ s^-1^ in leaves of seedlings growing in different P concentrations provided from different water-soluble fertilizer forms. These changes in MR reflect the redox states of P700+PC. Globally, the kinetics of the normalized MR suggest that the initial oxidation (the decreasing phase) followed by the re-reduction of P700 and PC (the increasing phase) and the two phases are separated by a transitory state, with similar rates of oxidation re-reduction rates of P700 and PC (MR_min_). Our results also showed alterations both in the amplitude and the rate of photoinduced changes of MR in P-deficient wheat seedlings compared to plants growing in sufficient P. At the beginning of treatment (S1 Fig), kinetic changes in the 820 nm modulated reflection showed equal re-reduction of P700 and PC, and this happened after 20 ms, except in plants growing in nutritive solution with Ortho-A and sufficient P for which P700 and PC were re-reduced after 16 ms. After 6 weeks of treatment (Fig 5B), we observed that re-reduction of P700 and PC occurred after 14 ms in all plants grown without P.

### Shoot phosphorus content

**Fig 6:**
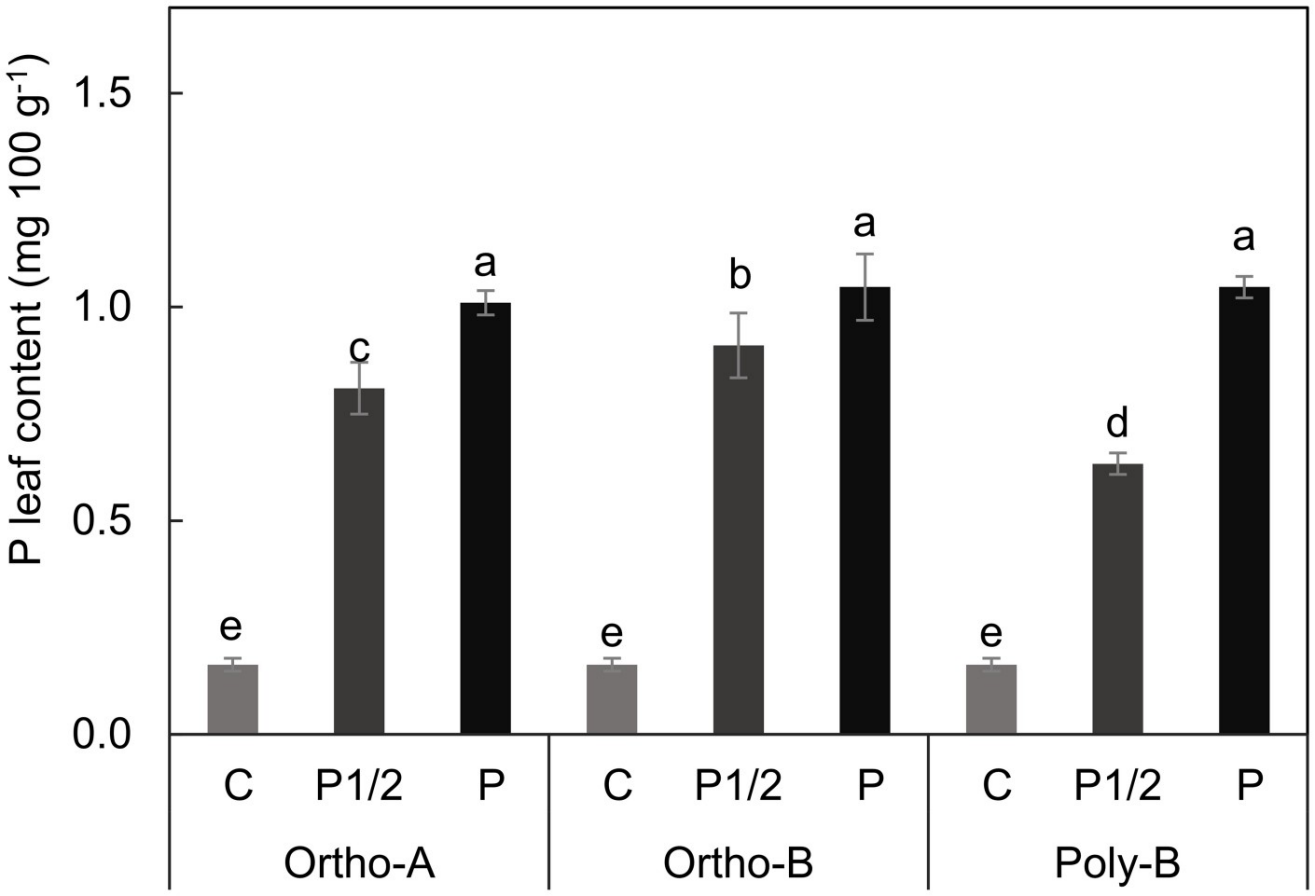
Phosphorus concentration in wheat plants grown in different P treatments for 6 weeks. The hydroponic solution was supplied with deficient (C and P1/2) and sufficient P (P) concentrations provided from different water-soluble fertilizers forms.

The results of our quantitative analysis of the P content in the leaves of wheat plants grown 6 weeks in hydroponic culture is presented in Fig 6. The ANOVA analysis showed an interactive effect between the studied factors. Regardless of the fertilizer form, plants grown in sufficient P showed the higher and significant P leaves content. However, under the P deficient treatment (P1/2), the leaves contained less P, and significant differences were observed between plants fertilized with different fertilizer forms. Plants grown in P1/2 provided by the Ortho-B form showed the highest P content. In comparison, plants grown in nutritive solution without P (Control) absorbed 0,16 mg.100 g^-1^, which had the least amount of absorbed P compared to the other treatments.

### Fluorescence and MR at 820 nm parameters

**Fig 7:**
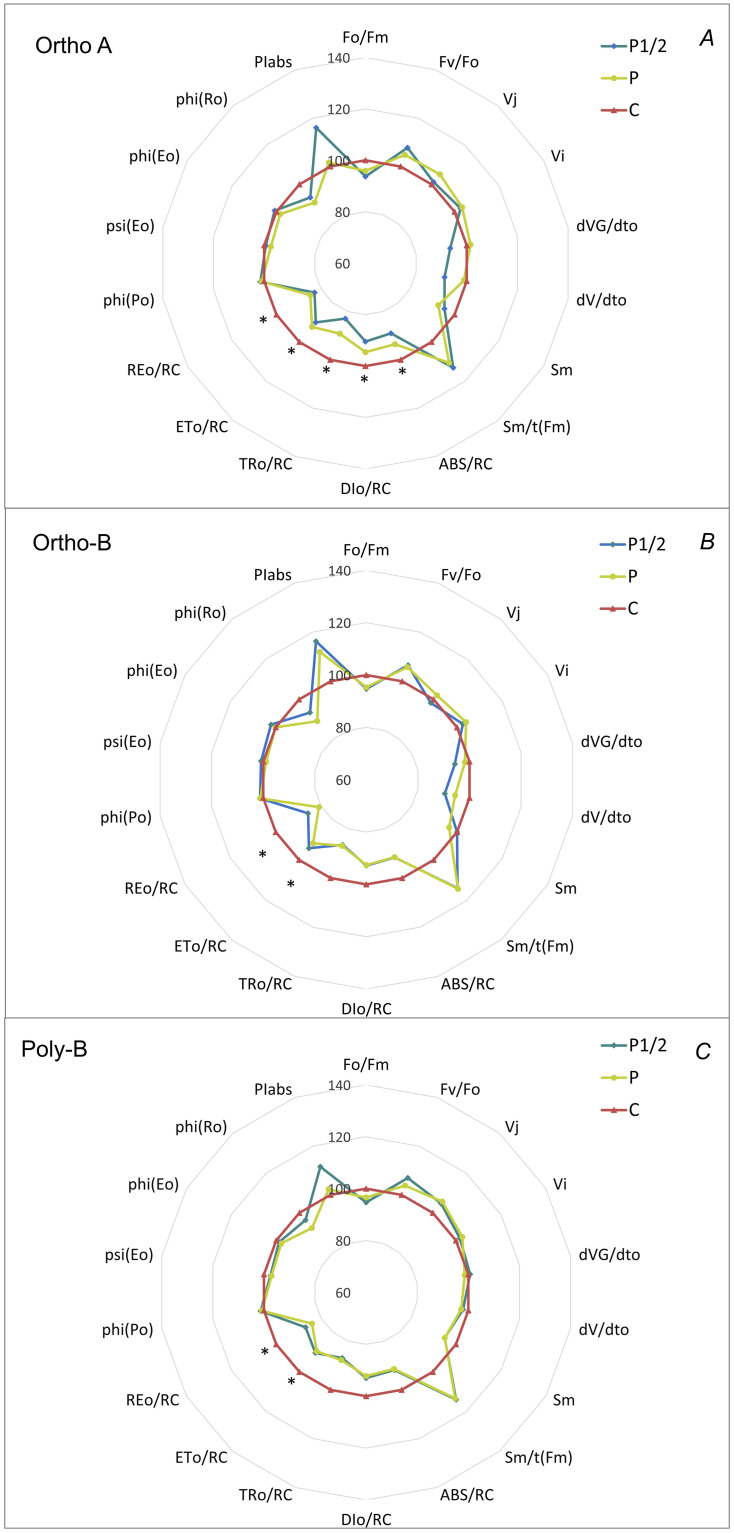
Radar plots of changes in JIP test parameters normalized to the values of the control treatment. (A) Ortho-A fertilizer form, (B) Orth-B, and (C) Poly-B. For each fertilizer form, means of fluorescence parameters marked by an asterisk differ significantly (p < 0.05, n = 10).

To understand better the effect of P deficiency on the chlorophyll a fluorescence transient, quantitative fluorescence parameters were normalized to the control treatment for each fertilizer form and presented in radar plots. Regardless of the fertilizer form, P deficient treatment increased significantly the parameters related to specific energy fluxes (i.e., per reaction center) such as electron transport in an active RC (ETo/RC) and electron transport beyond PSI (REo/RC) (Fig 7). Additionally, P deficient plants grown in ortho-A fertilizer form had higher values of dissipated energy flux and absorption flux per reaction center, represented by DIo/RC and ABS/RC, respectively (Fig 7A).

**Fig 8:**
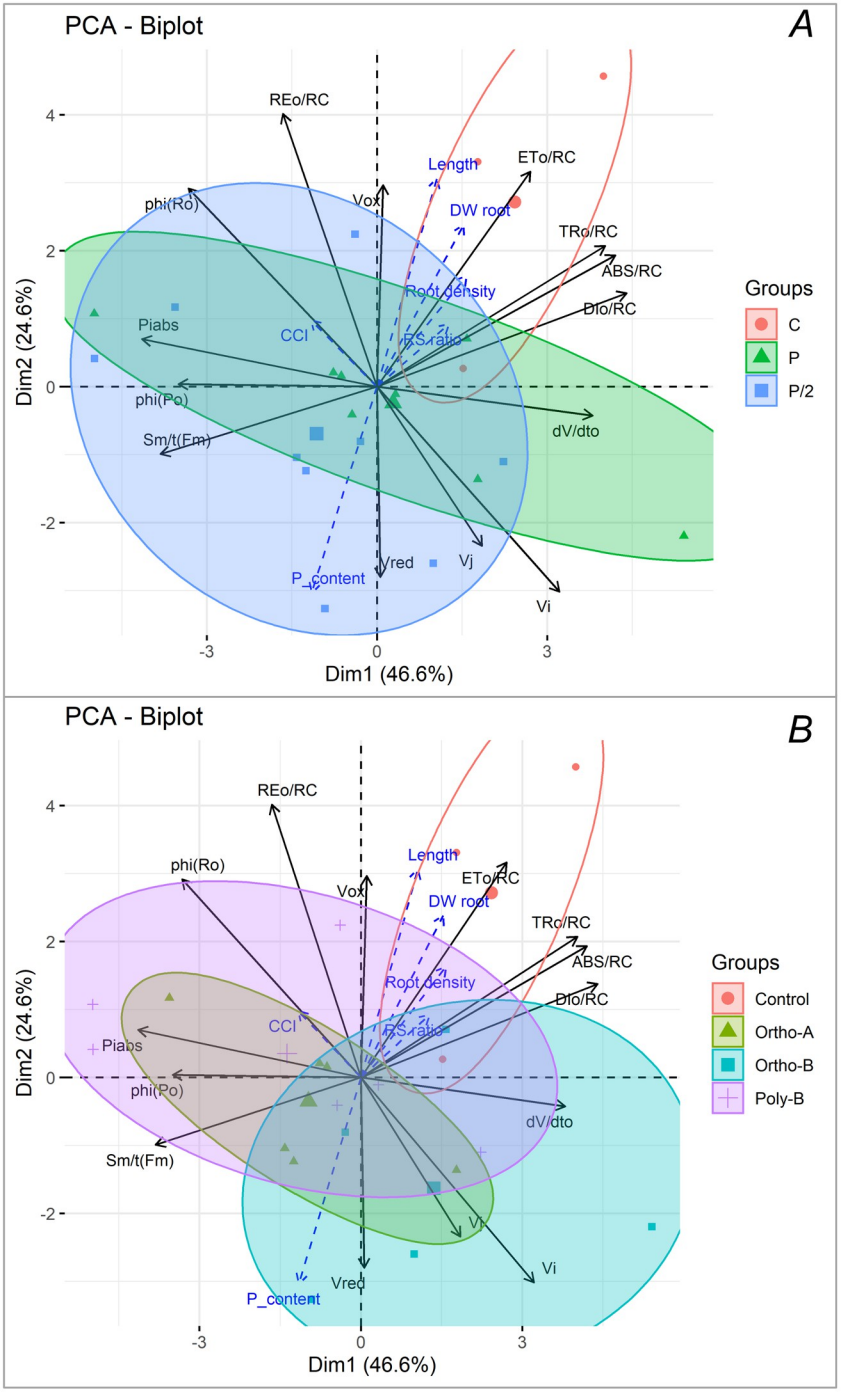
The principal component analysis biplot showing the first two variables of the principal component analysis. The black arrows represent the investigated parameters, and the size of the line is the weight parameter to define the principal component. The blue dashed arrows represent the supplementary variables. The marks represent the replicates colored by (A) the P level (C, 1/2P, and P) and (B) by fertilizer form. The big marks represent the barycenter of each P level.

Principal component analysis (PCA) was performed to highlight the relationship between the studied variables (Fig 8). Results showed that the first two components (PC1 and PC2) explained 46.6% and 24.6% of the overall variance, respectively, which is over 71% of the variability. PC1 was highly and positively loaded by the fluorescence parameters including dV/dto, ABS/RC, DIo/RC, TRo/RC, and V_I_ and negatively correlated to Sm/t(Fm), Phi(Po), Phi(Ro), and PI_abs_. On the other hand, MR parameters ν_red_ and ν_ox_ determined the variation in PC2, in addition to REo/RC and ETo/RC. The loadings are presented in S2 Table. The PCA-biplot revealed that plants grown in the absence of P have high values of root parameters, parameters related to the energy fluxes per reaction center, and oxidation rate. Increasing P supply has increased the re-reduction rate of P700 and PC and the relative fluorescence at the I and J steps, Chlorophyll content index was positively and highly correlated to the quantum yield for reduction of end electron acceptors at the PSI acceptor side (φ_Ro_).

## Discussion

In this study, we focused on some specific morphological and physiological adaptation processes and how combining chlorophyll *a* fluorescence transient and modulated reflection at 820 nm can enhance P deficiency detection.

### The impact of P deficiencies on morphological traits

According to previous research, the root system of P-deficient plants can undergo morphological and architectural changes to optimize P uptake. A preferential allocation of biomass to the roots, leading to an increased root/shoot ratio, was also described as a response to P deficiency [36–40]. Similar trends were reported in this study for plants experiencing P deficiencies (Fig 2B and 2D). As shown in Fig 3, our results suggest that plants grown in P-deficient solutions have altered root length and tips. Furthermore, our results confirm that the soluble fertilizer form also plays a role in determining the root morphology system. This might be explained by differences in the hydrolysis of water-soluble fertilizer forms that allowed P to be more available to root and then to shoot. In a recent study, it has been reported that water-soluble forms of fertilizer affected chickpea P uptake capacity [41]. In hydroponic conditions, Bessa et al. [42] observed that the P concentration in the plant tissues was positively correlated with root length, volume, and root dry mass of Cagaita plant. Several investigations have shown that changes of the root architectural system was a genotypic adaptation to P deficiency to allow a more efficient P uptake and to facilitates P absorption [43–45]. In accordance with these studies, it appears that Ortho-A makes P more available for plants, as confirmed by the P content absorbed compared to other treatments, even at a low content of P in the nutritive solution.

### The impact of P deficiencies on photosynthetic activity

It is well known that plant development and plant production is dependent upon net photosynthesis. Therefore, nutrients that affect photosynthesis, such as P, should also affect plant growth and development [37]. In our study, the decrease in stomatal conductance observed under P-deficient treatment might have undoubtedly induced a decrease in gas exchange. It has been reported that the regulation of stomatal opening and closure has a direct influence on the reduction of transpiration and solute transport through the plants [46]. We therefore notice that plants experiencing P deficiency modulate their stomatal conductance to optimise the rate of CO_2_ and water loss, to further preserve the energy used in photochemical phase in photosynthesis.

Regardless of the form of the water-soluble fertilizers, the effect of P deficiency on wheat depended on the concentration of P in the nutrient solution. In our experiment, after 6 weeks of treatment, the I-P phase was strongly affected (Fig 4A and 4B). The I-to-P rise was shown to be influenced by the electron transfer through cytochrome b6f to the PSI acceptor side [47]. Our results confirm previous data on the effect of P deficiency on IP phase of the OJIP transient [48, 49]. Furthermore, ΔV_IP_ was selected as one of the most important parameter to characterize the variability of plant photosynthetic efficiency [50]. The I-P phase has been used as an indicator to measure the PSI activity and the decrease of the I-P amplitude has been linked to a decrease of the content of PSI reaction centers [51]. On the other hand, it was reported that the I-P phase amplitude is independent of PSI activity since the low content of photo-oxidizable PSI did not result in a decrease in the amplitude of variable fluorescence between I and P steps [51]. The lost of the sigmoidal shape of the I-P phase may result from the regulation of rate of ATP and NADPH consumption in the Calvin cycle, which is affected indirectly by stromal orthophosphate and triose-P levels of the Calvin cycle [15]. P deficiency slow down the rate of ATP synthase, protons will accumulate in the thylakoid lumen, producing acidification, and the oxidation of the plastoquinone (PQH2) pool at the cytochrome b6f complex in the electron transport chain would eventually slow down. The I-step shape reflects how the reduced oxidation will alter the flow of electrons towards photosystem I (PSI) [52]. Also, increased I level at low P in our study (Fig 4C) indicates a slower electron flow to the PSI acceptors [53]. Furthermore, we suppose that P deficiency can alter the rate of NADPH and ATP consumption in the Calvin cycle. In a recent study, it was proposed that adequate P fertilization enhances NADPH consumption in the Calvin cycle [54].

The emergence of ΔI-band in the differential chlorophyll fluorescence curves normalized between O and P (ΔV_t_) might be linked to the damaged PSII’s acceptor side more severely than its donor side [55]. We suppose that the decreases of ΔV_t_ during the I-P phase reflects a decrease in the efficiency with which an electron is transferred to the PSI acceptor side. Ceppi et al. [56] suggest that relative changes in the PSI-content of the leaf can be effectively inferred from changes in IP-amplitude. The H-band that was observed after 6 weeks of growth without P fertilization (Fig 5A) were previously revealed in the fluorescence rise in PEG-6000 drought-stressed barley plants [57]. The H-band was attributed to an early activation of FNR in these plants [58]. Following a PSI fluorescence model, Lazár suggests this band, which appeared after the I step, reflects a PSI variable fluorescence [59]. It has been also linked to the reduction and oxidation of the plastoquinone pool (PQ, PC, and Cyt) and its size [60]. A positive H-band in P deficient leaves resulted from the higher reduction of PQ pool and the accumulation of reduced PQ electron carriers, which results a decrease in its capacity. Positive amplitudes of the difference in variable fluorescence curves were usually linked to nutrient deficiency [14]. On the other hand, leaves exhibited negative amplitudes of ΔV_t_ and the amplitude is more prominent under high deficiency treatment (control) than in moderate P deficiency treatment (P1/2) (S1A Fig). This was attributed to a slower reduction of the PQ pool in plants under high P deficiency compared to P1/2 treatment [61].

When exposed to phosphorus deficiency, parameters related to the energy fluxes per reaction center, namely ETo/RC, REo/RC, ABS/RC, and DIo/RC, were increased. These flux ratios are influenced by the active/inactive RCs, as the numbers of active centers decreased the two ratios increased [62]. The photons absorption and energy dissipation per reaction center increased with a decreasing P leaves content. Decreasing phosphorus level might affects the function of reaction centers, or the number of reaction centers in the PSII. In a previous study, the authors attributed the reduction in the number of reaction centers to the lower Fo values in a benthic diatom strain [63]. When studying the effect of P on electron transport chain of barley plants, phosphorus deficiency was found to reduce the PSII quantum yield, linear electron flow, and the fraction of open reaction centers [52]. An increase of ABS/RC and TR_o_/RC has been also observed by Meng et al. [37] while studying the phosphorus deficiency effects on photosynthetic performance in *Citrus grandis* leaves. The increase of the energy trapped by leaves per reaction center under phosphorus deficiency was attributed to the decrease of PSII active reaction centers or an increase in antenna size [19]. Nitrogen deficiency increased the antenna per active PSII RC (ABS/RC) and increased DIo/RC, as a consequence of the inactivation of some PSII reaction centers [64]. The quantum yield for reduction of end electron acceptors at the PSI acceptor side (φ_Ro_) was correlated to the first PC and showed a good relationship with chlorophyll content index. The strong correlation between fluorescence parameters, namely PSII quantum yield and efficiency, was explained by the fact that energy absorption and fluorescence emission are dependent on chlorophyll molecules [65]. Unfortunately, the data related to the effect of phosphorus on the modulated reflection at 820 nm is limited. In our study, the effect of P was observed starting from the steady phase (MR_min_) to the re-reduction phase (ν_red_) (Fig 5B). The P-deficient plants had the highest values of MR_min_ and MR_max_ at the sixth week after transplantation. P deficiency decreased ν_red_, indicating a slower re-reduction of P_700+_ and a slower PSI photochemical activity, which is the result of slower electron donation from P680. Similar result were reported by Feng et al. [24]; nitrogen deficiency has increased the MR_min_ of canola plants while increasing nitrogen N level increased the ν_red_ and ν_ox_. The increase of MR_min_ and the decrease of ν_ox_ has been linked to the slow oxidation rates of P700 and PC and the reduction of PSI photochemical activity [66]. In contrast to the previous study, our findings didn’t confirm the decrease of the ν_ox_ parameter at either of the measurement times, which was the result of the decline of the photosynthetic activity. Dąbrowski et al. [67] also found no decrease in the oxidation rate under the drought stress. It has been shown that only high stress may significantly impact the photoinduced variations in the P700 redox state in the fast phase, where the oxidation occurs [33].

## Conclusion

Results presented in this study confirm the idea that changes in root morphology and biomass translocation in P-deficient wheat seedlings are key factors that improve P uptake. The alteration in the electron transport chain, as a specific response to P deficiency was reported in the I-P phase. The lost of sigmoidal form within the I-P phase in P-deficient plants may be considered as an early-acting P deprivation signal. In this study we have attempted to explore the relationship between photosynthetic performance and P uptake in P-deficient plants. We found out that combining prompt fluorescence (PF) and modified reflection (MR) signals and including their derived parameters in a PCA analysis explained over 71% of the variation in the data using only two components and 75% using three components. Furthermore, P deficiency increased parameters related to the energy fluxes per reaction center by decreasing the number of active centers. Plants grown in high deficiency had also the highest values of MR_min_ and MR_max_ and the lowest values of ν_red_ due to slow re-reduction rates of P700 and PC. The re-reduction phase of MR_t_/MR_o_ occurs mainly during the I-P phase of chlorophyll fluorescence transient, which has been affected also by P deficiency. The results indicates that the two photosystems were fully synchronised and matched. However, the changes in the photosynthetic apparatus were more relevant in high phosphorus deficiency. Thus, it is difficult to detect changes in plants with low phosphorus deficiencies via the simultaneous measurements of prompt fluorescence and 820 nm modulated reflection.

## Supporting information

S1 Fig Changes in differential chlorophyll fluorescence curves modulated reflection at 820 nm. (A) Differential chlorophyll fluorescence curves normalized between O and P (ΔV_t_) and (B) changes in modulated reflection at 820 nm in leaves of seedlings grown for 1 week in different P concentrations and fertilizer forms. ΔV_t_ were calculated by subtracting the double normalized fluorescence values between F_O_ and F_M_ (V_t_), measured in plants growing in sufficient P treatment from those recorded in plants growing in low P concentrations (C and P1/2) using the formula ΔV_t_ = V_t (Control and P1/2)_ -V_t(P)_.
(TIF)

S1 Table The description of JIP-test and modulated reflection parameters (Strasser et al. 2010). (DOCX)

S2 Table Principal component analysis (PCA) loadings. (DOCX)
