# Supplementary figures and images for: PCA-based detection of phosphorous deficiency in wheat plants using prompt fluorescence and 820 nm modulated reflection signals
Source: PLoS One. 2023 May 24;18(5):e0286046. doi: 10.1371/journal.pone.0286046 (PMC10208481; doi:10.1371/journal.pone.0286046)

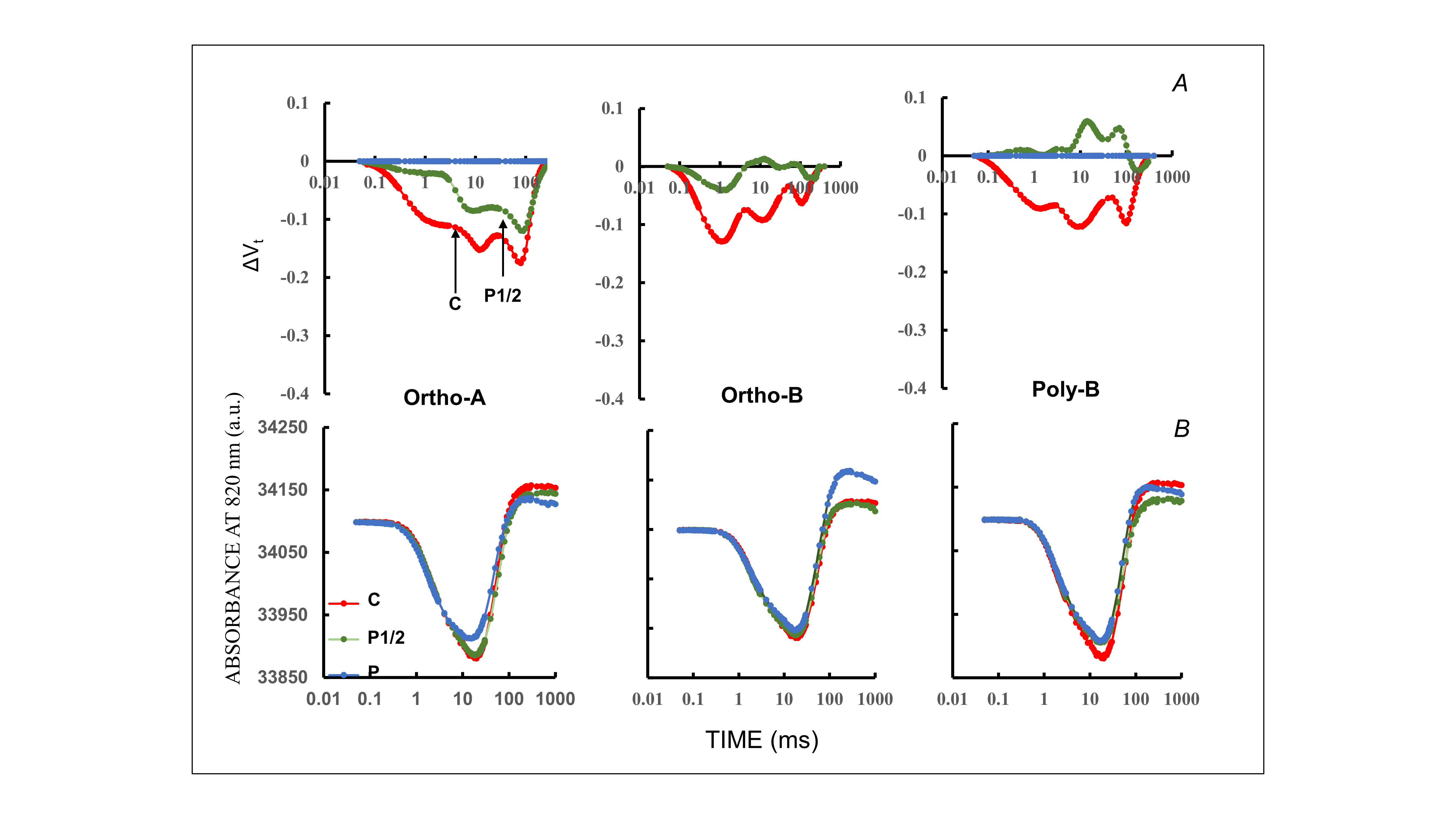

Supplement: S1 Fig — (A) Differential chlorophyll fluorescence curves normalized between O and P (ΔVt) and (B) changes in modulated reflection at 820 nm in leaves of seedlings grown for 1 week in different P concentrations and fertilizer forms. ΔVt were calculated by subtracting the double normalized fluorescence values between FO and FM (Vt), measured in plants growing in sufficient P treatment from those recorded in plants growing in low P concentrations (C and P1/2) using the formula ΔVt = Vt (Control and P1/2) -Vt(P). (TIF) [file pone.0286046.s001.tif]
