# Supplementary material for: PCA-based detection of phosphorous deficiency in wheat plants using prompt fluorescence and 820 nm modulated reflection signals
Source: PLoS One. 2023 May 24;18(5):e0286046. doi: 10.1371/journal.pone.0286046 (PMC10208481; doi:10.1371/journal.pone.0286046)
Supplement: S1 Table — (DOCX) [file pone.0286046.s002.docx]

| Parameter | Definition |
| --- | --- |
| F_v_/F_o_ | maximum ratio of quantum yields of SII |
| V_J_ | Relative fluorescence value at J-step [2ms] |
| V_I_ | Relative fluorescence value at I-step [30ms] |
| dVG/dto | Excitation energy transfer between the reaction centers (RC) |
| dV/dto | Rate of the reaction centers closure. |
| Sm | Normalized total area above the curve |
| S_m_/t(F_m_) | The average excitation energy of open RCs measured from time 0 to t(FM), the time required to achieve entire RC closure. |
| ABS/RC | Absorption flux (of antenna Chls) per RC |
| DI_o_/RC | Dissipated energy flux per RC (at t = 0) |
| TRo.RC | Maximum trapped excite on flux per active PSII reaction center |
| ETo.RC | Electron per active PSII reaction center |
| RE_o_/RC | Electron transport beyond PSI |
| ϕ_Po_ | Quantum yield of photosystem II photochemistry (Trapping): reduction of Pheophytin and Q_A._ |
| ϕ_Ro_ | Quantum yield for the reduction of end electron acceptors at the PSI acceptor side |
| ϕ_Eo_ | Quantum yield of electron transport to the plastoquinone pool |
| ψ_Eo_ | Probability that an electron moves further than Q_A_^-^ |
| PIabs | Performance index (potential) for energy conservation from photons absorbed by PSII to the reduction of intersystem electron acceptors |
| MR_min_ | Minimum of modulated 820 nm reflection intensity measured measured at 20 ms (steady state) |
| MR_max_ | Maximum of modulated 820 nm reflection intensity measured at 300 ms |
| ν_ox_ | Oxidation rate of P700 and PC |
| ν_red_ | Reduction rate of P700 and PC |

**S1 Table. The description of JIP-test and modulated reflection parameters (Strasser et al. 2010)**
