# Supplementary material for: PCA-based detection of phosphorous deficiency in wheat plants using prompt fluorescence and 820 nm modulated reflection signals
Source: PLoS One. 2023 May 24;18(5):e0286046. doi: 10.1371/journal.pone.0286046 (PMC10208481; doi:10.1371/journal.pone.0286046)
Supplement: S2 Table — (DOCX) [file pone.0286046.s003.docx]

**S2 Table. Principal component analysis (PCA) loadings**

|  | PC.1 | PC.2 |
| --- | --- | --- |
| Vj | 0.391 | -0.494 |
| Vi | 0.679 | -0.636 |
| dV/dto | 0.802 | -0.089 |
| Sm/t(Fm) | -0.805 | -0.209 |
| ABS/RC | 0.887 | 0.408 |
| DIo/RC | 0.929 | 0.291 |
| TRo/RC | 0.849 | 0.437 |
| ETo/RC | 0.571 | 0.668 |
| REo/RC | -0.350 | 0.847 |
| ϕ_Po_ | -0.738 | 0.008 |
| ϕ_Ro_ | -0.701 | 0.615 |
| PIabs | -0.874 | 0.148 |
| ν_ox_ | 0.023 | 0.626 |
| ν_red_ | 0.013 | -0.590 |
